# What works to recruit general practices to trials? A rapid review

**DOI:** 10.12688/hrbopenres.13650.1

**Published:** 2023-02-10

**Authors:** Daire Buckley, Sheena M. McHugh, Fiona Riordan

**Affiliations:** 1 School of Public Health, University College Cork, Cork, Ireland

**Keywords:** Primary health care, general practice, recruitment strategies, randomised controlled trials

## Abstract

**Background**: Recruitment challenges are a barrier to the conduct of trials in general practice, yet little is known about which recruitment strategies work best to recruit practices for randomised controlled trials (RCTs). We aimed to describe the types of strategies used to recruit general practices for trials and synthesize any available evidence of effectiveness.

**Methods: **We conducted a rapid evidence review in line with guidance from Tricco *et al*. Eligible studies reported or evaluated any strategy to improve practice recruitment to participate in clinical or implementation RCTs. PubMed, Embase, and Cochrane Central Library were searched from inception to June 22 ^nd^, 2021. Reference lists of included studies were screened. Data were synthesized narratively.

**Results: **Over 9,162 articles were identified, and 19 studies included. Most (n=13, 66.7%) used a single recruitment strategy. The most common strategies were: in-person practice meetings/visits by the research team (n=12, 63.2%); phone calls (n=10, 52.6%); financial incentives (n=9, 47.4%); personalised emails (n=7, 36.8%) or letters (n=6, 52.6%) (as opposed to email ‘blasts’ or generic letters); targeting practices that participated in previous studies or with which the team had existing links (n=6, 31.6%) or targeting of practices within an existing practice or research network (n=6, 31.6%).  Three studies reporting recruitment rates >80%, used strategies such as invitation letters with a follow-up phone call to non-responders, presentations by the principal investigator and study coordinator, or in-person meetings with practices with an existing affiliation with the University or research team.

**Conclusions: **Few studies directly compared recruitment approaches making it difficult to draw conclusions about their comparative effectiveness. However, the role of more personalised letter/email, in-person, or phone contact, and capitalising on existing relationships appears important. Further work is needed to standardise how recruitment methods are reported and to directly compare different recruitment strategies within one study **. **

**PROSPERO registration:** CRD42021268140 (15/08/2021)

## Introduction

As the first point of contact in the provision of services, general practice is a cornerstone of primary care services in both the prevention and management of ill health ^1^. With primary care forming a fundamental part of health service reform worldwide, the need to conduct high-quality clinical trials in the general practice setting is essential to guide quality improvements, underpin service delivery, and translate it to best-practice for patient care ^2, 3^.

Trials conducted in general practices are becoming more common, in particular, cluster and pragmatic trials ^4– 8^ The latter puts more emphasis on the generalisability of findings to real-world clinical practice ^7^. Cluster randomised trials are often used to test the effectiveness of quality improvement interventions and implementation strategies at the practice level, targeting patients via professionals and/or targeting professionals directly. However, recruitment of practices and physicians to practice-based studies can vary greatly ^9^ which has implications for the representativeness of the findings. Despite a growing focus on trials in general practice, recruitment of practices to participate in research is a major challenge. Factors commonly reported to positively influence recruitment include clinician interest in the research topic ^10– 15^ and rapport between the practice and research team ^11– 13^. Barriers include an inability to commit time or perception that the research is overly time-consuming ^3, 13– 19^ and a lack of space or infrastructure ^2^. Little is known about which recruitment strategies may work best to recruit practices for trials ^12^.

Reviews to date have focused on methods to recruit *patients* to trials in general practice ^20, 21^, rather than methods to recruit *practices*. For example, in a 2018 systematic review of strategies to improve recruitment to randomised trials, of 68 eligible studies ^22^ only one tested an approach (mailed postcard teaser campaign) to recruit practices for a clinical trial ^23^. Moreover, while there is a substantial body of literature on strategies to reach ‘hard to reach’ population subgroups (e.g., ethnic minorities, vulnerable or isolated adults) ^24– 26^, the concept of the ‘hard to reach’ practice is less well defined ^27^ and it is unclear how to actively target them.

To address these gaps, the current rapid review aims to synthesise the available evidence on strategies used to recruit general practices for trials. The findings will provide a useful resource for researchers conducting trials in general practice. This review also aimed to extract information on the profile of practices, and, where possible, narratively examine the association between the strategy and participant profile. This will provide researchers with an insight into the profile of ‘hard to reach’ practices and may inform future recruitment strategies within general practice trials.

## Methods

We conducted a rapid evidence review of trials in general practices which report or test recruitment strategies. We conducted this review in line with the guidance developed by Tricco *et al*. ^6^ and the Preferred Reporting Items for Systematic Reviews (PRISMA) ^28^. The review was prospectively registered on the PROSPERO database (CRD42021268140) on 15 ^th^ August 2021.

### Eligibility criteria

***Types of interventions.*** Eligible studies were those that reported or tested any strategy to recruit general practices to participate in a randomised controlled trial (RCT). Examples of strategies could include invitation letters, endorsement, and financial incentives for participating practices. Studies reporting strategies to recruit practices by recruiting physicians were also included.

***Types of studies.*** Eligible studies included any RCT, be they clinical trials or implementation trials (trials of implementation strategies) ^6^. Both full scale RCTs and pilot RCTs ^29^ were eligible for inclusion. Observational, cross-sectional, and qualitative studies were excluded. Studies which only reported strategies to recruit *patients* through general practices were excluded, as were strategies to recruit primary care *physicians only*.

Only studies that mentioned recruitment in the title or abstract were eligible for inclusion. Both studies that *specifically* focused on recruitment within the trial and studies that simply reported their strategy as part of the description of the main trial (but recruitment was not the focus of the study) were eligible. Studies were not excluded on the basis of the design used to evaluate recruitment strategies; this could be qualitative or quantitative.

Hypothetical trials were excluded, i.e., studies that ask potential participants whether they would take part in a trial if it was run, but the trial does not exist. Studies focusing on recruitment of healthcare organisations other than general practices, were excluded.

***Types of articles.*** Only peer-reviewed papers in academic journals were considered eligible. Protocol papers and conference abstracts were excluded. Selection of relevant papers was restricted to English-language publications only.

***Types of outcomes.*** Primary outcomes were the proportion of eligible practices recruited and the time taken to recruit practices. Secondary outcomes included author reflections on the effectiveness of the recruitment strategy and whether it was successful or not, and the cost of the strategy. Studies had to report one of the primary outcomes to be included in the review. The full inclusion and exclusion criteria are summarised below in Table 1.

**Table 1.** Eligibility criteria.

	Inclusion criteria	Exclusion criteria
**Study**	Clinical, implementation or pilot RCTs Studies were not excluded on the basis of the design used to test recruitment strategies.	Observational, cross-sectional studies (case-control/ cohort) Qualitative studies
**Types of articles**	Peer-reviewed papers in academic journals	Non-peer reviewed sources (e.g., reports) Conference abstracts Protocol papers
**Population**	Primary care practices, family practices, general practices	Other primary care or community-based services. e.g., Public Health Nursing, Community Pharmacies, Dentists and Optometrists.
**Intervention**	Strategy to recruit practices to the trial. Strategy to recruit practices via recruiting physicians.	Strategy to recruit *patients* through general practices. Strategy to recruit general practice *physicians*.
**Outcome**	**Primary outcomes:** • Number of practices responding to, or recruited by, a certain strategy • Time to recruit practices. **Secondary outcomes:** • Authors reflections on the success or challenges of the strategies employed. • Cost of the recruitment strategy.	Primary outcomes of the recruitment strategy are not reported.

### Information sources

The following electronic databases were searched for relevant studies: PubMed (1946 to present), Embase (1947 to present), and Cochrane Central Library. All searches were conducted on the 22 ^nd^ of June 2021. The search strategies used a combination of text words and relevant indexing, related to trials, recruitment, and general practices. The full search strategy is provided as extended data ^30^. Reference lists of included studies were also screened.

### Study selection

Articles were retrieved from databases and imported to Mendeley reference management software for de-duplication. Articles were imported into Rayyan for screening. Approaches to rapid reviews can include using one person for screening with verification of a subset of records by another ^31^. Titles and abstracts of identified articles were screened by one reviewer (DB) with independent screening of 25% of the articles by a second reviewer (FR). Each full text was screened independently by two reviewers (DB and FR). Disagreements were resolved through consensus or referral to a third reviewer (SMH).

### Data extraction and quality assessment

Using a standard form, DB extracted data on the study title, authors, the aim of the study, the year of publication, the country in which the study was carried out, the nature of the population, the nature of the study setting and the study design used to test the recruitment strategy (if applicable), details of the strategy and its effectiveness, the profile of recruited practices if provided (e.g., size, location, staff, and other descriptors used by the study authors), and any author reflections or descriptions of the strategy impact, and/or recruitment of ‘hard to reach’ practices. We did not apply a standard definition of ‘hard to reach’. Practices were considered ‘hard to reach’ if defined as such by the author. A second reviewer extracted the data in duplicate (FR). Authors were contacted for additional data if required. Any disagreements were discussed and resolved via referral to a third reviewer (SMH) to achieve consensus. 

We did not undertake a quality assessment since the aim was to characterise practice recruitment strategies and describe the outcomes of those strategies. 

### Data synthesis

A narrative synthesis approach was used to combine and summarise the findings from included studies. Given the wide range of strategies, we summarised the findings from the five most common strategies. Before completing the review, we expected that the results would not be standardised in a way that permitted meaningful pooling to undertake a meta-analysis. Furthermore, given the short timeline within which to conduct the review (June to September 2021), we did not anticipate it would be feasible to combine quantitative results in a meta-analysis. Strategies were organised into categories through discussion between DB and FR. The categories were informed by preliminary searches of the literature, and existing studies of recruitment ^2, 22^. We also categorised the overarching recruitment *approach*. Recruitment approaches which used multiple strategies in one phase were referred to as ‘multiple strategy approach’ whereas those which staggered one or more strategies were referred to as ‘single staggered approach’. Where possible, the strategy types were compared in terms of the profile of practices recruited to explore potential links/patterns between the strategy and participant profile.

## Results

### Study overview

**Figure 1.**
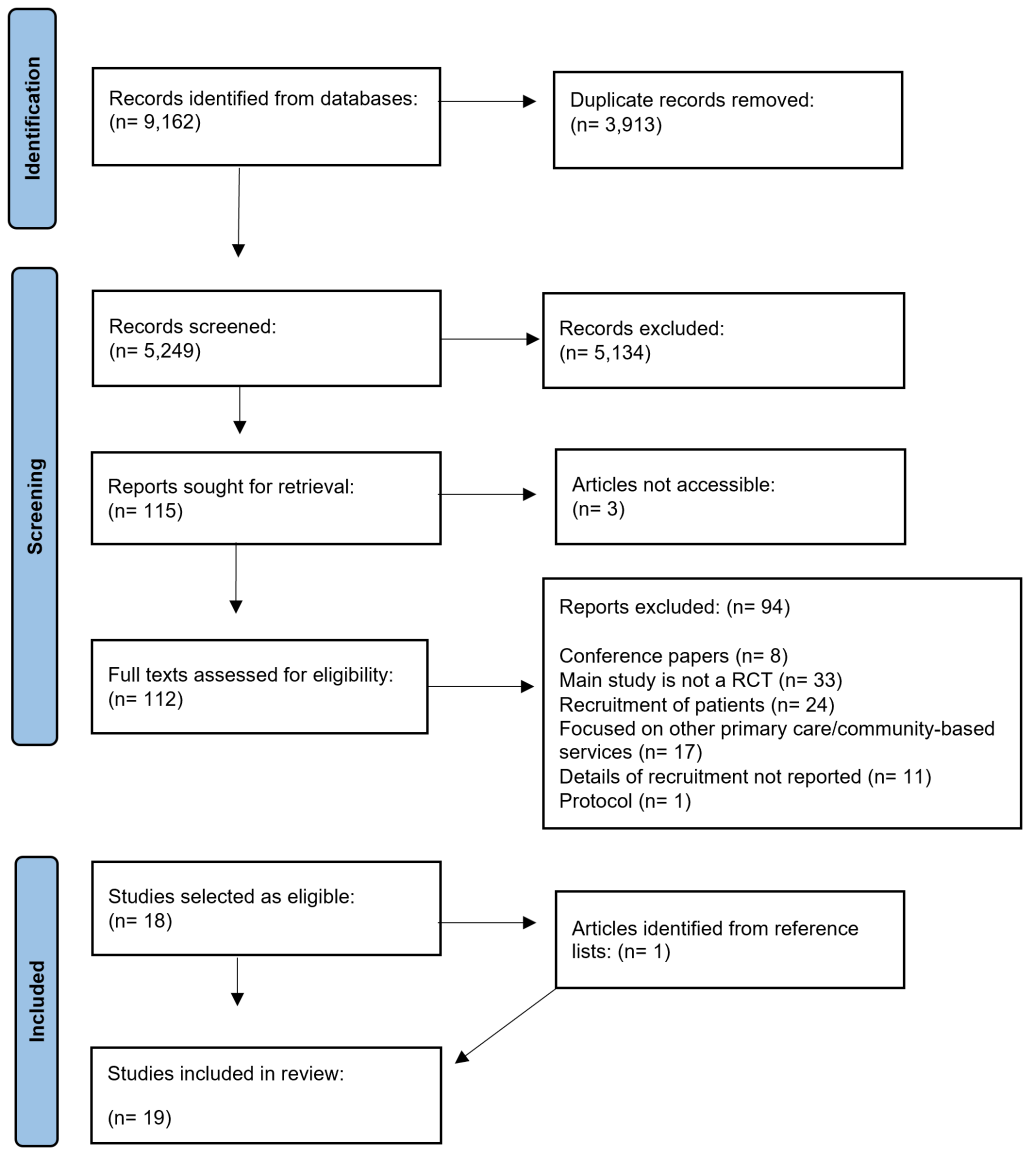
PRISMA flow diagram.

From 9,162 articles identified from three databases, 112 full texts were screened for eligibility based on inclusion and exclusion criteria, of which 18 were included ( Figure 1). One additional study was identified from reference lists. Therefore, a total of 19 studies were included in the review, predominantly from the US (n=6; 31.6%) ^32– 37^ and Australia (n=6; 31.6%) ^11, 14, 23, 38– 40^, with other regions in Europe (Ireland: n=1; 5.3%) ^16^ and Germany: n=1; 5.3%) ^41^, the UK (n=4; 21.1%) ^42– 44^ and England and Wales (1; 5.3%) ^45^ also represented. Most studies were published after 2010 (n=12, 63.2%).

Most studies (n=18) did not formally evaluate the effect of recruitment strategies, these studies descriptively reported the outcome of the recruitment strategy or strategies. One study by Lee *et al.* ^23^ evaluated recruitment strategies through a two-arm RCT embedded in a clinical trial.

### Recruitment strategies

Overall, 13 studies (66.7%) ^13, 14, 16, 32, 35, 36, 38, 40, 41– 45^ used one or more strategy staggered across different phases to reach a certain group of practices and did not distinguish the outcome of individual strategies within the approach (‘single arm, staggered approach’). The remaining studies used multiple recruitment approaches in one phase (‘multiple strategy approach’) comprising one or more strategies, sometimes comparing the outcomes of each approach separately ^34^. Where possible, the strategy types were compared in terms of practice profile recruited to infer potential associations between the strategy and participant profile.

The most common strategies used were:

(1) in-person meetings/visits by members of the research team with practices (n=12, 63.2%);
(2) phone calls to practices (n=10, 52.6%);
(3) use of financial incentives (n=9, 47.4%);
(4) personalised emails (n=7, 36.8%) or letters (n=6, 52.6%) (as opposed to an email ‘blast’ or generic letters);
(5) targeting of practices which were participants in previous studies or with which the team had existing links (n=6, 31.6%) or targeting of practices within an existing practice or research network (n=6, 31.6%).

The most common combination was personalised letter and phone calls (n=5, 26.3%) or in person visits and phone calls (n=3, 15.8%) or a combination of all three (n=2, 10.5%). The least common strategies reported (only used by one study) were: mass distribution of recruitment materials at professional meetings or conferences, magazine editorials, flyers distributed via patients, project webpage, clinical audit activity, informational videos, and information cards. The full list of strategies is provided in Table 2.

**Table 2.** Recruitment strategies used by included studies (n=19).

Study	Total using this strategy	Curtan, *et al.* ^32^	Cuthel *et al.* ^33^	Down, *et al.* ^42^	Ellis, *et al.* ^34^	Gágyor, *et al.* ^41^	Gulliford, *et al.* ^43^	Horspool, *et al.* ^45^	Leathem, *et al.* ^16^	Lee, *et al.* ^23^	Reid, *et al.* ^38^	Tan, *et al.* ^11^	Flanagan, *et al.* ^44^	Fletcher, *et al.* ^39^	Loskutova *et al.* ^35^	Ruud, *et al.* ^36^	Reed, *et al.* ^46^	McBride, *et al.* ^37^	Perkins, *et al.* ^14^	Dormandy, *et al.* ^13^
**Overall approach**	N	S	M	S	M	S	S	S	S	M	S	M	S	M	S	S	S	M	S	S
**Strategies**																				
**Using an existing ** **practice network**	6	X	X				X					X			X				X	
**Previous links ** **with practices**	6		X		X	X								X		X	X			
**Presentations** at professional meetings, conferences	3				X							X		X						
**Distribution** at professional meetings, conferences	1				X															
**Magazine editorial**	1				X															
**Professional ** **newsletter**	2				X							X								
**Interest survey**	2				X							X								
**Trial endorsement**	5				X		X											X	X	X
**Flyers via patients**	1											X								
**Webpage**	1											X								
**Clinical audit**	1											X								
**Webinar**	3	X	X												X					
**In-person visits**	12	X	X		X	X			X		X	X		X		X	X	X		X
**Informational ** **video**	1	X																		
**Incentive**	9	X	X			X			X	X				X	X		X			X
**CPD credits**	3	X	X							X										
**Faxed letter ** **(generic)**	0																			
**Faxed letter ** **(personalised)**	2				X							X								
**Letter (generic)**	2			X	X															
**Letter ** **(personalised)**	10					X		X	X	X	X	X	X		X			X		X
**Email ** **(generic)**	1		X																	
**Email (personalised)**	7		X		X		X	X				X		X	X					
**Phone calls**	10		X	X	X			X	X	X			X		X			X		X
**Postcard**	2									X					X					
**Information card**	1														X					
**Reminder**	5		X	X			X	X							X					
**Cold calls**	3		X		X				X											
**Total strategies**			**6**	**11**	**3**	**13**	**4**	**4**	**4**	**5**	**5**	**2**	**11**	**2**	**5**	**9**	**2**	**3**	**4**	**2**

Abbreviations: S, Staggered approach; M, Multiple strategy approach

### In-person meetings

Among the 12 studies which included in-person visits or meetings at practices as a recruitment strategy, meetings were typically conducted by member(s) of the research team ^36, 39^ or the team *and* Principal Investigator (PI) ^32, 40^. Some specified that a clinical member (e.g., research nurse or GP researcher) of the team conducted the visits ^16, 38, 41^ while others did not provide this detail ^11, 13, 33, 34, 37^. Four studies involved conducting in-person meetings with practices with which some of which the team already had links ^32, 36, 39, 41^.

In terms of timing, most visits were made to practices which expressed an interest *after* initial letters/emails of invitation or cold calls ^13, 16, 36– 39, 41^, presentations at conferences ^39^ or after a webinar delivered to practices within a research network after initial outreach by the medical director ^32^. Ellis *et al.*, made visits to practice groups after obtaining permission from medical directors of a regional network of primary care practices. Some studies provided more detail on the format and content of these visits ^11, 16, 37, 38^ ( Table 3).

**Table 3.** Description of practice visits.

Study	Who	Content/focus	Timing/Frequency
**Tan *et al.*** ^11^	Not specified	• Emphasised the significance or value of research topic • Explained randomisation process (practice level) • Highlighted benefits of the trial intervention, such as accredited GP activities • Highlighted clinical resources, and a list of service providers for referral. • Provided details of financial incentive (AUD$100) • Outlined the simplicity of research logistics	After informal contact established (via presentation, flyers, mail outs, newsletters, or survey) One visit Time not specified.
**Leathem *et al.*** ^16^	Research nurse	• Provided the opportunity for practitioners to ask questions • Gathered needs/requirements • Collected practice data (staffing information, computerisation, and special interests)	After contact established via phone call and letter with information sheet One visit At time convenient to the practice e.g., over lunchtime
**McBride *et al.*** ^37^	Not specified	• Introduced study details • Introduced practice to project personnel • Assessed practice environment	After letter and follow up phone call. One visit At time convenient to the practice e.g., over lunchtime; food provided
**Reid *et al.*** ^38^	Regional Medical Coordinator * or Research nurse	• Provided additional materials or tools i.e., video, receptionist information, subject flow chart, waiting room promotional poster and a study protocol • Ensured suitable space available for trial • Medical education session on management of hypertension • Discussed participant recruitment and study protocol	After mail out and expression of interest received Two visits and (at some sites) a dinner meeting

*A practising GP was employed on a part-time basis as a regional medical coordinator. The RMC had academic status within the host department of general practice and was primarily responsible for the conduct of the study in the respective state

### Phone calls

Most of the nine studies which used phone calls provided no further detail on what the call involved. In some studies, the phone call was the initial ^16^ or primary ^42^ contact mode used, in others, phone calls were part of a suite of strategies ^34, 35, 37^, or used in a final round of recruitment, to provide a ‘boost’ after letter/email invitations ^23, 44, 45^.

Two studies explicitly mentioned making “cold calls” to practice managers ^16, 33^ identifying practices within a certain radius of the study region, from telephone books, medical society' membership list and provider directories ^34^. Randomly ordered lists of practices were used to confirm practice eligibility and ascertain their initial *interest* ^16^ Leathem *et al.* provided more detail on the content of the calls, that they included a brief explanation and mention of the financial resource, and that they avoided making these calls at especially busy times such as Monday mornings and Friday afternoons and asked reception staff for appropriate times to speak to a GP or practice manager, not wanting to use time slots reserved for patients.

### Personalised mails or letters

Seven studies used emails ^11, 33– 35, 39, 43, 45^, ten used letters ^11, 16, 23, 34, 35, 37, 38, 42, 44, 45^ and three used a combination of both ^11, 35, 45^. Four studies described issuing reminders using letters or emails ^33, 35, 43, 45^. No definition of what was considered a *personalised* or *personal* email or letter was provided by the studies which used these strategies but based on the study context this was taken to be emails/letters which were addressed to a specific named person in the practice as opposed to the practice more generally. For example, one study reported using personalised emails along with generic email ‘blasts’ to mailing lists ^33^.

Five of the thirteen studies emailed or mailed practices within an existing network ^11, 33, 43^, or used existing contacts ^32, 34, 41^, sometimes relying on individuals within those networks to circulate the emails ^34, 43^ Three described using opinion leaders ^34^, or local clinicians or organisations ^38^ endorsing the study by sending the letters ^34, 38^, or using the letterhead of the local sponsoring organization, along with signature from a study physician ^37^. Where a network or existing contacts were not used, different groups were targeted: GP academics ^11^, GP attendees of a professional forum ^11^; practices in an existing network database supplemented with publicly available addresses from business directories ^11, 37^.

Four of thirteen studies described the content of the email or letter, including key features of the trial ^23, 45^, expected requirements ^16, 23, 45^ and projected workload ^16^, and financial and CPD compensations for participation ^23^, role of the sponsor ^38^, rationale for the study ^38, 45^, and information about the study funder ^38, 45^.

Where a network or existing contacts were not used, different ‘pools’ or groups were targeted: GP academics ^11^, GP attendees of an aged care forum ^11^; practices in an existing network database supplemented with publicly available addresses from business directories ^11, 37^.

### Financial incentives

Nine studies mentioned using a financial incentive as a recruitment strategy ^13, 16, 23, 32, 33, 35, 39, 41, 46^, seven specifying the scale of the ^13, 16, 32, 33^ practice fee ranging from $4,500 ^33^ to €1,000/£700 ^16^) or fee per patient (€100 per completely documented patient ^41^, $50AUD for eligible participant. ^23, 39^).

### Existing networks and links

Six studies targeted practices with which they had existing links or had involved in a previous study ^33, 34, 36, 39, 41, 46^, six targeted practices within an existing practice or research network ^11, 14, 32, 33, 35, 43^. One did both, targeting practices in an existing primary care network, but also starting with sites that had engaged in previous projects to minimize the number of “cold calls” ^33^.

## Outcomes

### Quantitative

Outcomes reported were yield as a proportion of the total practices *targeted* (n= 18, 94.7%) ^11, 16, 23, 32– 39, 41– 46^, yield as a proportion of practices who *expressed an interest* (n=4, 21.1%) ^34, 36, 38, 44^, time taken for recruitment (n=6, 31.6%) ^11, 16, 35, 37, 42, 45^ and cost (n=3, 15.8%) ^23, 34, 37^.

***Yield.*** The reported yield (% of practices recruited of those targeted) ranged from 0.40% ^34^ to 83% ^44, 46^, with an average response of 46.8% (SD = 24.8). Ellis *et al.* ^34^ compared 10 different strategies; reporting the lowest yield (0.40%) cumulatively from six opt-in marketing tactics [i.e., mass advertising, conference distribution, mass fax, direct mail, opinion leader email, in-person presentation] (n=53/13,290) compared to 51% (n=90/176) from a survey-based strategy. This involved adding survey items to gauge interest in the study into an annual network survey of primary care providers, and then following up with practices who indicated an interest. Of the six opt out strategies, they reported mass advertising (published article and medical society inserts), an opinion leader email, and medical conference distribution were the least effective strategies. 

Studies that reported recruitment rates over 80% ^36, 44, 46^, aimed to recruit smaller numbers of practices. They issued invitation letters with a follow-up phone call to non-responders ^44^, or conduced presentations by the PI and study coordinator ^46^ to, or in-person meetings with ^36^, practices with an existing affiliation with the University or research team.

*Time taken to recruit*

Six studies reported the time taken to recruit practices ^11, 16, 35, 37, 42, 45^ which ranged from 12 months (recruitment target: 48 practices) ^16^ to 1.5 months (recruitment target: 27 practices) ^11^. One study reported the number of contacts ^45^; reporting the total number of times practices were contacted from the initial invitation (post or email or phone call) to randomisation as 6.8 (SD = 3.5), and the mean number of contacts required to gain an expression of interest (EOI) from the initial postal contact invitation to randomisation as 3.01 (SD = 1.6). More detail on recruitment time is provided in Table 4.

**Table 4.** Time take to recruit practices.

Study	Strategies	N practices recruited	Time
Down *et al.* ^42^	1. Phone (including reminders) 2. Mailed information to those who expressed an interest	169	Mean number of days to agree to participate 213 (7 months from initial contact).
Leathem, *et al.* ^16^	1. Cold calls 2. Mailed information to interested practices 3. In-person visit.	48	12 months
Tan, *et al.* ^11^	1. Mailouts 2. An online survey 3. 5-15-Minute presentations to practices and within the university department 4. Word of mouth through professional networks 5. Promotion at a medication workshop 6. Emails to attendees at an aged care forum and to GP academics 7. Newsletters 8. Study webpage 9. Promotion of accredited clinical audit activity which formed part of the trial	75	1.5 months (range: 0.5–3.5 months) for a practice visit to be organised from the point of contact
Loskutova *et al.* ^35^	1. Information card 2. 1-Hour informational webinar 3. Recruitment letter	25	Average time to enrol practices from the beginning of recruitment until completion of all required enrolment paperwork: 71 calendar days (range 11–107 days) Average time for initial acceptance or refusal by the organisation: 30 days
McBride *et al.* ^37^	1. Recruitment via physicians at managed care organisation using organisation letterhead 2. Direct mail to practice leaders 3. Direct mail to physicians	14	8-months for strategy 3 5 or 7 months for strategy 2 4-months for strategy 1

*Cost*

Three studies reported the cost of the recruitment strategies ^23, 34, 37^ which ranged from $6,471.69 to $41,340 for the postcard teaser ^34^ to $3,834 for a direct mail to physicians ^36^. Comparisons were not valuable given the variation in approach and different health systems.

*Comparison*

Four studies ^11, 23, 34, 37^, narratively compared the outcomes of different recruitment approaches but did not use a formal evaluation design. Two compared the response rate (RR) (i.e., % *responded* to the approach) ^23, 37^ one compared the recruitment yield (RY) (i.e., % *recruited* by using the approach), and one reported both RR and RY ^34^.

Lee *et al.* ^23^ compared the response ( *any response* to the mailout, positive or negative) to a standard mailout approach (personalised invitation letters followed by a max of three phone calls) to a standard approach preceded by a postcard teaser campaign involving two postcards with different short phrases, one without affiliations, one with logos and affiliations sent to practices. They reported a RR of 5.9% (n = 11/186) in the Teaser Campaign group and 7.5% (n = 14/186) in the Standard Mail group, reporting the former did not increase the odds of a response to the subsequent letter (OR = 1.18, CI = 0.75–1.85, p = 0.49).

Tan *et al.* ^11^ used eight approaches reporting the recruitment *yield* of each approach: mailout to GPs in network database supplemented by publicly available business directories (RY = 0.7% , 9/1322), an online survey (RY =0.4% , 6/1400,), presentations at practices (RY = 28%, 5/18), word of mouth within professional network (RY = 30%, 3/10 professionals making informal contact were recruited, but the total contacted is unknown), medication management workshop (RY = 14.2% , 2/14), University Department of GP presentation (RY = 100%, 1/1 of those making informal contact, total attending = 30-50), contact with the practice via a third party to relay trial information and promote the trial while offering a clinical audit activity (a quality improvement process for continuing GP professional development) (RY = 100%, 1/1 making informal contact were recruited, but total contacted is unknown), and newsletters and email invites (RY = 0%, total contacted/reached is unknown).

Ellis *et al.* ^34^ reported a RR of 0.4% (53/13290), to the six opt-in marketing strategies they employed: mass advertising (medical society inserts RR: 0.04%; 2/5350; published article RR: 0%; 0/3500)), conference (RR: 0.94% (1/106)), mass fax distribution (RR:1.13%; 44/3882), minority provider (RR:1.25%; 4/319), opinion leader (RR:1.02%; 1/98), in-person provider presentation (RR: 2.86%; 1/35),

About a third (RR:34%; 18/53) of responders to the marketing strategies (1-6) were recruited to participate in the study. The approaches that recruited the most practices per effort and the most cost-effective approaches were in-person meetings (RR:41.7%; 5/12), followed by building on previous relationships (RR:33.3%; 9/27) and borrowing from established networks (RR:10.8%; 19/176).

Finally, McBride *et al.* ^37^ used three approaches, direct mail to physicians with return postcards (RR = 3.6%, 90/2485), recruitment via physicians at managed care organisations using the organisation letterhead (RR =25.5%, 11/43), and direct mail to practice leaders (RR =61.3%, 27/44).

### Profile of recruited practices

Few studies provided a detailed profile description, and where they did, the characteristics reported varied. Seven studies ^11, 13, 16, 32, 34, 38, 42^ reported the profile of practices which had been recruited. Based on the limited detail available, smaller proportions (<50%) of recruited practices appeared to be single-handed (ranging from 16% to 33% of recruited practices) ^16, 34, 38, 42^, practices with a predominantly female GP workforce (34%) ^34^, and based in urban areas (9% ^38^ and 22% ^32^).

The percentage of single-handed practices recruited, compared across five studies (using different strategies) varied: 16% ^42^, 18% ^11^, 19% ^46^, 28% ^38^, and 33% ^16^. Tan *et al.* reflected that medium-sized practices (69% of practices recruited) were easier to recruit quickly than large practices, attributing this to the availability of a key person who could act as a facilitator (e.g. practice manager or nurse).

Two studies compared the profile of recruited practices to practices which were not recruited ^13, 42^ and neither reported significant differences in practice profiles. Down *et al.*, who provided the most detailed comparison ^42^ reported weak associations with the staff gender proportions in the practices; recruited practices had a lower average proportion of male GPs, and were less likely to have all male GPs. However, overall, they reported there were no ‘convincing’ differences in staffing (number of GPs, % UK trained GPs, proportion of single-handed practices, proportion with only non-UK trained GPs) and list sizes, between practices who were recruited and those who were not.

### Qualitative

Five studies also included qualitative findings related to recruitment efforts ^11, 13, 33– 35^, including weekly debriefing notes from meetings between research staff and recruiters ^33^ or qualitative data from notes ^35^, recruiters’ online diary entries ^33^, administrative data ^35^, communication and recruitment material ^35^, a survey ^34^ and semi-structured interviews with providers/practice staff ^13, 33^. Though not a primary aim of this review, of note, these studies reflected on barriers (out-of-date information about the practice ^33^; lack of engagement with recruiters within the primary care network ^33^, competing practice priorities ^33^ and lost paperwork ^35^) and facilitators (perceived importance of the research topic ^13, 34^, and a general interest in research ^13, 34^) of recruitment.

Two authors reflected in more detail on the perceived pros and cons or challenges associated with deploying a strategy. One felt that although they were building on existing relationship with practices by going through a network, supplementary recruitment strategies were still often required to engage sites, including additional personal calls and site visits ^33^. Tan *et al.* ^11^ felt that more successful strategies involved direct contact with GPs (individualised invitations, online GP surveys, and face-to-face presentations); in particular in person practice visits, despite costs, were key, providing time to build rapport with GPs and other practice staff, and from their perspective allowed them to engage practices who might not have otherwise been recruited. They felt that providing information by phone, “passing the message” by GPs, practice nurses or staff, or leaving written material for GPs was not a fruitful strategy. They reported GPs and practice staff being “overwhelmed” by emails and post. 

## Discussion

### Summary

This rapid review of strategies used to recruit primary care practices to trials yielded 19 studies. The most common strategies included in-person meetings/visits by members of the research team with phone calls to practices, financial incentives, personalised emails, or letters (as opposed to an email ‘blast’ or generic letters) and targeting practices which took part in previous studies or with which the team had existing links or were part of an existing practice or research network. Most studies used a single recruitment approach, be it using one strategy or layering different strategies. Six studies used multiple recruitment approaches at the same time, with three comparing the outcomes of the different approaches; suggesting direct mail to practice leaders compared favourably to direct mail to physicians ^37^ a postcard teaser did not have a significant impact on recruitment compared to standard mail out ^23^, and a combination of both opt-in and opt-out approaches yielded the most practices recruited per effort and proved to be the most cost effective ^34^. Studies reporting the highest recruitment yield (>80% of practices contacted were recruited) ^36, 44, 46^ used invitation letters with a follow-up phone call to non-responders ^44^, presentations or in-person meetings with practices which had an existing affiliation with the University or research team ^36, 46^.

### Implications

Two factors made it challenging to draw conclusions about the most effective recruitment strategies from the included studies. First, the level of detail provided on recruitment strategies varied across all studies. All studies bar two specifically focused on recruitment within the trial (i.e., specific aim to describe or evaluate recruitment). These two studies just reported their strategy as part of the description of the main trial ^41, 43^. Often studies provided very limited detail on the types of strategy and the yield. The CONSORT checklist includes reporting recruitment and follow-up period (items 14a and b) ^47^ and additional guidance on the reporting of embedded methodological studies (to test interventions to improve recruitment to trials) and highlights the need for sufficient details (how, where and when) to allow replication of recruitment interventions ^47^. The success of practice recruitment approaches has important implications for trial conduct, including the length of time engaged in recruitment and cost, and the types of practices and subsequently patients included in the trial.

The second factor making it challenging to draw conclusions from this review was that only six studies ^11, 23, 33, 34, 37, 39^ directly compared strategies and reported the outcomes of each strategy. There was limited information available to determine the relative strengths of a strategy. Only one study tested the effectiveness of strategies ^16^. While there has been a drive for trialists to embed evaluations of recruitment strategies within their trials ^48^, this work has largely focused on strategies to recruit patients rather than practices ^22^. A systematic review of studies to date indicates that effective strategies for patients include follow up phone calls after non-response to mailed invitation, and informing people what they will be receiving in the trial. How to optimise recruitment strategies to target *general practices* is unknown. For example, some studies used endorsement as a strategy, using clinicians at different points to communicate with practices. It would be helpful to understand whether certain messengers or messages are more effective than others.

With a lack of standardised reporting of practice recruitment strategies and their effectiveness, knowledge of what works (and does not work) is not shared across studies. Capturing this type of information is increasingly important. The number of trials based in general practice is growing both internationally ^49, 50^ and in Ireland ^51^; with the establishment of the Primary Care Network Ireland, there is potentially a wealth of information among teams in Ireland about what works well in terms of recruitment, yet this is not systematically captured or shared.

In spite of the limitations of poorer reporting and the lack of studies of strategy effectiveness, there are three key lessons arising from this review. First, while there appears to be some potential benefit from capitalising on existing relationships and networks, it is important to consider how to supplement this approach to ensure a diverse range of practices are recruited. This was an issue flagged by Carr *et al.* in their discussion piece on practice readiness to engage in research. The authors emphasized the need to identify and ‘optimise’ recruitment approaches which can appeal to clinicians in general practices which do not have existing links to networks or universities and may therefore tend to be missed for research opportunities ^27^. By supporting development of relationships between practices and researchers, primary care networks can play an invaluable role in in supporting recruitment for larger scale clinical trials ^52^.

Second, informing potential participants about the direct benefits the study offers is important to encourage recruitment ^52^. The use of incentives was a common strategy, both financial and CPD, and these incentives were flagged in early communication with the practices. Ward *et al.*, in their practical guide on recruitment, advise determining the schedule of payments in advance, and whether there will be a flat rate, payment for fee-for-service, or combination of both methods used ^52^ – both approaches were used in the identified studies. However, the level of incentive varied between studies, making it difficult to know what financial compensation is acceptable and the level which would affect recruitment. The level of incentive will also depend on the nature of the trial, what is required of practices and for how long. Future studies could consider using experimental designs (e.g., discrete choice experiments) to elicit practice preferences. Studies evaluating a more standardised approach to incentives are needed to provide more robust evidence regarding the use of financial or CPD incentives. 

Third, certain recruitment approaches reported very limited yield, often broad mailouts or marketing campaigns ^11, 34^ However, it was not easy or possible to determine a denominator in these instances. While approaches such as targeting practices known the study team or from previous studies may have delivered a higher yield, it is important to note they were targeting much smaller pool of practices. It is important to consider whether such an approach can ensure a diverse range of practices; those less accustomed to participating in research which may be less ‘research ready’ may not be reached ^27^. Trialists should consider the potential value (and costs) of approaches which cast a ‘wider net’ (e.g., like the mass marketing approach used by Ellis *et al.*), versus more targeted approaches. To better understand their value, it would be helpful if trialists documented and compared the practices identified and recruited through these means to determine whether they lead to recruitment of a different profile of practice compared to those recruited through other more direct approaches.

### Limitations

To our knowledge this is the first study to synthesise the evidence on strategies used to recruit general practices for trials. However, there are some limitations. As this was a rapid review, to identify and include studies we relied on recruitment being mentioned in the title or abstract. Ideally a review would identify and include all trials conducted in primary care and review the methods section to gather extra details on recruitment. While we followed best practice guidance for rapid reviews ^31^, we did reduce the number of screeners at the title and abstract screening stage and searched a limited number of databases. Lastly, we did not undertake a quality assessment since the aim was to describe outcomes of strategies – not all studies specifically tested strategies.

## Conclusion

The purpose of this rapid review was to synthesise the available evidence on strategies used to recruit general practices for trials. However, we found descriptions of recruitment approaches were limited and very few studies in this review directly compared strategies or reported on the outcomes of the strategies they used for recruitment. This made drawing conclusions difficult. However, certain strategies were more common (in-person meetings/visits by members of the research team with practices, personalised letters/emails, phone calls, financial incentives, and capitalising on existing relationships or within an existing practice or research network), and some did appear to offer a greater yield (invitation letters with a follow-up phone call to non-responders, presentations or in-person practice meetings with practices with an existing affiliation with the research team). We suggest future research could focus on evaluating these strategies in embedded trials, determining how to optimise them. While no tactic alone may solve the challenges in terms of clinical trial recruitment, it is possible, the strategies outlined in this review could contribute to better recruitment and result in more diversity in clinical trials. Lastly, it is important to standardise how recruitment strategies are reported to consolidate learning across trials and determine what truly works to recruit general practices.

## Data Availability

All data underlying the results are available as part of the article and no additional source data are required. Zenodo: What works to recruit general practices to trials? A rapid review. PubMed search strategy. https://doi.org/10.5281/zenodo.7271269 ^30^. Zenodo: PRISMA checklist for’ What works to recruit general practices to trials? A rapid review’. https://doi.org/10.5281/zenodo.7277893 ^28^. Data are available under the terms of the Creative Commons Attribution 4.0 International license (CC-BY 4.0).
