# Supplementary material for: What works to recruit general practices to trials? A rapid review
Source: HRB Open Res. 2023 Feb 10;6:13. [Version 1] doi: 10.12688/hrbopenres.13650.1 (PMC10518848; doi:10.12688/hrbopenres.13650.1)
Supplement: Peer review history

# Reviewer report: Reviewer response for version 1

**DOI:** 10.21956/hrbopenres.14927.r35754

**Published:** 2023-09-13

**Authors:** Elisavet Moutzouri

**Affiliations:** 1 University of Bern, Bern, Switzerland

This rapid evidence review aimed to explore the strategies employed  from clinical trialists in order to successfully recruit general practices in clinical research. This is an interesting topic.  This review adds considerable evidence in a domain with little available literature till today, which however is important and evolving. The article is well written and is throughout compehensive. Still, there are some small points for the authors to address.

 Introduction: introduction is well written and underscores the important message the study want to transmit. The authors could also refer to the fact that recruiting in GP practices could reduce the underrepresentation of special groups in clinical research like multimorbid elderly people ^1^). 

 Methods: clearly describe, Table 1: remove "Studies were not excluded on the basis of the design used to test recruitment strategies": not needed in the table

 Data synthesis: please describe the calculation of "yield".

 Results: Table 2: Considering listing columns according to strategies most frequently used.

 Financial incentives: possible to expand and describe in more detail as well as if some of this strategies had more impact?

 Outcomes: "studies that reported recruitment rates over 80% ^36, 44, 46^, aimed to recruit smaller numbers of practices." : what "smaller number" exactly means? 

 It would be useful in one of the tables to be able to see how large these studies (how many patients) were as also the medical domain they involved and how many GP Practices were recruited.

 Discussion --> Implications:  "All studies bar two specifically focused on recruitment within the trial": check grammatical errors

Are the rationale for, and objectives of, the Systematic Review clearly stated?

Yes

Is the statistical analysis and its interpretation appropriate?

Yes

Are sufficient details of the methods and analysis provided to allow replication by others?

Yes

Are the conclusions drawn adequately supported by the results presented in the review?

Yes

Reviewer Expertise:

Lipids, multimorbidity

I confirm that I have read this submission and believe that I have an appropriate level of expertise to confirm that it is of an acceptable scientific standard.

## References

[1] : Lipid-Lowering Trials Are Not Representative of Patients Managed in Clinical Practice: A Systematic Review and Meta-Analysis of Exclusion Criteria. *J Am Heart Assoc* .2023;12(1) : 10.1161/JAHA.122.026551 e026551 10.1161/JAHA.122.026551 36565207PMC9973576

# Reviewer report: Reviewer response for version 1

**DOI:** 10.21956/hrbopenres.14927.r35756

**Published:** 2023-08-31

**Authors:** Nan Kennedy

**Affiliations:** 1 Vanderbilt University Medical Center, Nashville, USA

This paper represents a rapid evidence review of the literature on recruiting GP practices to clinical trials. Recruitment strategies were examined for the 19 studies considered eligible for review. While the sample size did not allow for many statistically valid comparisons among strategies to determine effectiveness, the authors were able to draw some useful general conclusions on the types of tactics being employed in recruiting practices and how well they tend to work.

 As clinical trial recruitment continues to be difficult and the involvement of practices has been shown to help substantially in increasing trial enrollment, this review is both timely and valuable. This reviewer especially lauds the article’s emphasis on the importance of ‘widening the net’ to invite practices that may not be a part of an existing research network to increase trial participant diversity. The authors acknowledge, and rightly so, that increasing the range and diversity of participating practices, although potentially more difficult and time-consuming to recruit, may be as useful a marker of overall success as cost and percentage yield.  

 The article is well-written with a logical flow and is easy to follow. The rationale for, and objectives of the review are clearly stated. Sufficient details in the Methods and Analysis are provided to enable replication. The statistical analysis, while necessarily limited, appears to be appropriate given the parameters of the study. The frank discussion of the study limitations was helpful. The implications and conclusions, while limited, are supported by the results. Overall, the paper provides a strong argument on the need for recruiting practices, the dearth of evidence about how best to go about it, the limited data on recruitment strategies currently available, and the necessity of future embedded trials to better evaluate these tactics.

 Several areas in the manuscript needed further clarification or detail:

- Authors should consider revising their terminology of ‘hard to reach’ populations or practices, which places the responsibility to be aware of and available for trials on the potential participants or practices, rather than recognizing that research teams are the ones who must bear the burden of finding ways to reach them (see added citations).
- Under the section on ‘Recruitment strategies,’ in the third paragraph, the second sentence reads, ‘The least common strategies reported (only used by one study)’ implies that the list of strategies that follows were all used by the same, single study, whereas they were evidently used by different studies. Consider revising the sentence to state: ‘The least common strategies reported (only used by one study *each*)…’
- ‘Opt in’ and ‘opt out’ strategies are mentioned, but without explanation. It would be helpful to define these terms.
- In the section on ‘Time taken to recruit,’ the timeframe of ‘initial invitation to randomisation’ was used. What or who is being randomised in this context? Perhaps it means from initial invitation to the randomisation of the first participant in a trial through that practice? If so, this should be made clear. The related table (Table 4) mentions nothing about randomisation. It would be helpful to make the text and table congruent with respect to what is being measured.

Are the rationale for, and objectives of, the Systematic Review clearly stated?

Yes

Is the statistical analysis and its interpretation appropriate?

I cannot comment. A qualified statistician is required.

Are sufficient details of the methods and analysis provided to allow replication by others?

Yes

Are the conclusions drawn adequately supported by the results presented in the review?

Yes

Reviewer Expertise:

Clinical trial recruitment consultant and research information specialist

I confirm that I have read this submission and believe that I have an appropriate level of expertise to confirm that it is of an acceptable scientific standard.

## References

[1] : Hard to reach? Language matters when describing populations underserved by health and social care research. *Public Health* .2022;205: 10.1016/j.puhe.2022.02.006 e28-e29 10.1016/j.puhe.2022.02.006 35282902

[2] : Inclusive research: Repositioning the. *J Adv Nurs* .2023;79(8) : 10.1111/jan.15555 2779-2781 10.1111/jan.15555 36541273

# Reviewer report: Reviewer response for version 1

**DOI:** 10.21956/hrbopenres.14927.r33906

**Published:** 2023-06-09

**Authors:** Stuart G Nicholls

**Affiliations:** 1 Ottawa Hospital Research Institute, Ottawa, Canada

**What works to recruit general practices to trials? A rapid review**

 The current review explores an important topic insofar as the recruitment of institutions to studies serves as a gateway to participant recruitment. Indeed, given the vast amount of research that is conducted in primary care it is surprising that such a limited amount of literature is available.

 That said, I do have several comments on the article that represent some underlying concerns.

 BACKGROUND

 The authors refer to ‘hard to reach’ groups and particularly the literature regarding recruitment of patients. Yet it isn’t clear how this terminology relates to general practice or primary care. While I appreciate that the authors here used definitions provided within individual articles, the rationale and goal of the authors is not clear in terms of what they see as the fundamental issue or who they see in this group.

 What was the rationale for a rapid review? As noted by Tricco, rapid reviews are useful in time-limited circumstances, such as emergencies or crisis. What was the time issue in this case (especially given publication is 2 years after the search was conducted)? A justification for a rapid (and more limited) review is needed beyond a similar scoping review. This is important given the limitations imposed on terms in the title and abstract and the lack of synonyms (e.g. enrolment, accrual etc.).

 Also of note, reference 6 is attributed to Tricco but is actually listed as Wolfenden et al. (2021) – Tricco is listed as a reference 31 in the references.

 METHODS

 It would be helpful to clarify eligibility for the included studies which seem to be studies that took part within the context of an RCT. However, the authors note that “observational, cross sectional and qualitative studies” were excluded. I think the scope needs clarifying as most studies would or could be described as observational, cross sectional, or qualitative insofar as they were themselves not RCTs, but took place within the context of an RCT.

 Is general practice universal term or structure for primary care? How was this defined and operationalised in terms of the inclusion or exclusion criteria? For example, was it synonymous with “family physician” which may be a term used in jurisdictions outside the UK and Ireland?

 The search is now 2 years old – can the authors comment on the impact this likely has on the results? This is particularly relevant given the rapid nature of the review (as opposed to a scoping review).

 For the screening, was any training or calibration undertaken?

 Following on from this: how common were disagreements? Was an a priori level of agreement set whereby additional double-review would be needed? It would seem inappropriate to only review 25% if a high proportion of those 25% resulted in disagreements and required a third reviewer.

 The authors state that “We did not undertake a quality assessment since the aim was to characterise practice recruitment strategies and describe the outcomes of those strategies.” This seems out of place given the aim is to assess the outcomes, surely one needs to know the quality of the study in order to assess how useful that outcome data was. Further, it is inconsistent with the PROSPERO registration information that states: “Risk of bias (quality) assessment Quality will be assessed using existing tools, for example the Cochrane Risk of Bias tool or the Critical Appraisal Skills Programme (CASP) checklist for RCTs or Joanna Briggs Institute Critical appraisal checklist for RCTs."

 The authors state “Furthermore, given the short timeline within which to conduct the review (June to September 2021), we did not anticipate it would be feasible to combine quantitative results in a meta-analysis.” Why was there such a short timeline?

 DISCUSSION

 The CONSORT checklist is discussed, but it may also be worth reflecting on the CONSORT extension for cluster RCTs, which includes provision for reporting on practices. It may be that the work there could be useful for the present context.

Are the rationale for, and objectives of, the Systematic Review clearly stated?

Partly

Is the statistical analysis and its interpretation appropriate?

I cannot comment. A qualified statistician is required.

Are sufficient details of the methods and analysis provided to allow replication by others?

No

Are the conclusions drawn adequately supported by the results presented in the review?

Yes

Reviewer Expertise:

patient engagement, health services research

I confirm that I have read this submission and believe that I have an appropriate level of expertise to state that I do not consider it to be of an acceptable scientific standard, for reasons outlined above.
